# The need for worldwide policy and action plans for rare diseases

**DOI:** 10.1111/j.1651-2227.2012.02705.x

**Published:** 2012-08

**Authors:** John Forman, Domenica Taruscio, Virginia A Llera, Luis A Barrera, Timothy R Coté, Catarina Edfjäll, Désirée Gavhed, Marlene E Haffner, Yukiko Nishimura, Manuel Posada, Erik Tambuyzer, Stephen C Groft, Jan-Inge Henter, on behalf of the International Conference for Rare Diseases and Orphan Drugs (ICORD)

**Affiliations:** 1 New Zealand Organisation for Rare DisordersWellington, New Zealand; 2 National Centre for Rare Diseases, Istituto Superiore di SanitàRome, Italy; 3 Fundación GEISERMendoza, Argentina; 4 Institute of Inborn Errors of Metabolism, Javeriana UniversityBogota, Colombia; 5 National Organization for Rare DisordersWashington, DC, USA; 6 Shire Human Genetic TherapiesShire AG, Eysins, Switzerland; 7 Childhood Cancer Research Unit, Department of Women's and Children's Health, Karolinska Institutet, Karolinska University Hospital SolnaStockholm, Sweden; 8 Haffner AssociatesRockville, MD, USA; 9 Promotion Research on Intellectual Property, The University of TokyoTokyo, Japan; 10 Research Institute for Rare Diseases, Instituto de Salud Carlos IIIMadrid, Spain; 11 ABConsultLeuven, Belgium; 12 Office of Rare Diseases Research, National Institutes of HealthBethesda, MD, USA

**Keywords:** Rare diseases, Orphan drugs, Public health

## Abstract

There are more than 6000 rare diseases (defined as affecting <5/10 000 individuals in Europe, <200 000 people in the United States). The rarity can create problems including: difficulties in obtaining timely, accurate diagnoses; lack of experienced healthcare providers; useful, reliable and timely information may be hard to find; research activities are less common; developing new medicines may not be economically feasible; treatments are sometimes very expensive; and in developing countries, the problems are compounded by other resource limitations. Emphasis is required to support appropriate research and development leading to better prevention, diagnosis and treatments of rare diseases. Notably, clinical trials using already existing drugs may result in new, affordable, treatment strategies. Moreover, rare diseases may teach us about common disorders.

**Conclusions:**

Countries are encouraged to implement specific research and development activities within their individual capabilities, so that patients worldwide have equal access to necessary interventions to maximize the potential of every individual.

## Introduction

There are more than 6000 rare diseases, currently defined as affecting <1 per 2000 individuals in Europe or <200 000 people in the United States (1). Many rare diseases are diagnosed at the age of childhood, making diagnostic awareness and knowledge on treatment and care particularly important for paediatricians. The rarity of these diseases can create special problems for affected populations including the following:

1. Difficulties in obtaining timely and accurate diagnoses.
2. Lack of experienced healthcare providers.
3. Useful, reliable and timely information may be hard to find.
4. Research activities are less common.
5. Developing new medicines may not be economically feasible.
6. Treatments are sometimes very expensive.
7. In developing countries, the problems are compounded by other resource limitations.

Rapidly expanding scientific and technological advances are greatly improving our ability to intervene in various health conditions, including rare diseases. In the US and EU, legislation including the Orphan Drug Act (1983) and the Orphan Regulation No 141/2000 has brought many rare disease treatments into clinical practice (1,2). Rare disease issues feature increasingly in US and EU policy papers, as the EU Council Recommendation on rare diseases (2009), new action plans (http://www.europlanproject.eu), the EU Committee of Experts on Rare Diseases reports on the state of art of rare disease policy and research, and the International Rare Disease Research Consortium. However, numerous challenges lay ahead (3). Many countries do not yet have policies for rare diseases and orphan products research and development.

The International Conference on Rare Diseases and Orphan Drugs (ICORD, http://www.icord.se) is a non-profit society, drawing together members from academia, patient advocacy, medicine regulatory, healthcare industry, healthcare services, and public policy agencies and organizations around the globe. Our mission is to improve health and welfare of patients with rare diseases and their families worldwide and reflect on rare disease policies for the future. We try to increase awareness and action internationally by using best practices and examples from all over the world and by bringing together top level experts from different stakeholders worldwide. We present this position statement as a basis for information to and discussion with national governments and international health bodies about rare disease policies.

## Health Priorities, and Legal, Ethical and Social Issues

The United Nations Universal Declaration of Human Rights (Article 25.1), the International Covenant on Economic, Social and Cultural Rights (Article 12.1) and the United Nations Convention on the Rights of the Child include important statements about rights to health care. Moral philosophy offers additional guidance. The ethical principle of Justice requires that the needs of rare disease populations are specifically addressed, as for any minority or underserved community. A global approach to rare diseases is needed to utilize the experiences and knowledge gained from rare diseases research and orphan products development.

Key principles for adoption in health policy include:

1. Rare diseases are a significant public health issue. Together they may affect up to 8% of the population, corresponding to a significant minority population.
2. Health care and treatment for rare diseases is a human rights issue. Non-discrimination, justice and equity of access to health care all require that specific policies are put in place to address the needs of people affected by rare diseases.
3. Every country is encouraged to have a rare diseases research development program, with emphasis adjusted to its existing capabilities.
4. A comprehensive approach to rare diseases is needed, including education, prevention, diagnosis, care and treatment, social support and inclusion as well as support of both basic and clinical research.
5. Quality information, informed consent and autonomous decision-making are critical for upholding the rights and protection of patients and their families. Combining genetic knowledge with screening to identify risks should be actively pursued to provide choices about prevention, balanced with careful attention to informed consent and autonomous decision-making.
6. Patient groups play an important role in the development of knowledge about rare diseases and are suggested to be included at all levels in the development of their policies and services.

## An Action Plan for Implementing Rare Diseases Policies

These twelve points are provided as guidance for the implementation of rare disease policies:

1. **Action plans**. Governments should recognize that rare diseases create disparities and vulnerabilities in health status for affected populations.
2. **Specific programs and policies**. Governments should recognize the human rights issues inherent in rare disease care and treatment across the lifespan. Specific programs and policies may be needed to protect those rights.
3. **Allocation of resources**. Governments should adopt policies for equitable allocation of resources towards all aspects of rare diseases, including information resources, basic research, clinical care, treatment development and clinical research. Moreover, support for clinical trials using already registered existing drugs and other treatments but for new indications should also be considered because this may be effective and cost-effective, as shown in many childhood cancer where remarkably improved outcomes have been achieved by repeated clinical trials using established drugs in new combinations (4).
4. **Specific counterbalancing policies**. Governments and health systems should offer incentives to encourage development of rare disease treatments and recognize problems with the research and development costs of such treatments. Regulatory requirements for clinical trials are important protections for patient safety, and it should be considered to approach these requirements differently for rare and very serious diseases.
5. **Cost effectiveness assessment should consider wider factors**. Health economics criteria, if used and if applicable, should not only consider the cost of treatment but also take into account personal, social and economic benefits of treating diseases.
6. **Specific benefits of research into rare diseases**. Research policies should note the specific benefits of research into rare diseases for gaining information, such as the cause of more common and multi-factorial diseases (5,6). This may justify weighting of research funds towards rare diseases.
7. **Recognition of gaining knowledge to aid prevention**. Gaining knowledge of disease processes may be as relevant for prevention as for treatment of a disease. Opportunities to prevent serious rare diseases should also be a research priority.
8. **Encouragement of industry to contribute to rare disease knowledge**. Industry should be encouraged to increase its ‘public good’ contributions to rare disease knowledge, such as through donation of products or techniques.
9. **Patient advocacy groups participation in advisory groups and expert panels**. Patient advocacy groups provide important information and support and should be independently involved in advisory groups and expert panels to consider specific policy, ethical controls, risk management and service planning (7).
10. **Development of information networks and support group capacity**. Good information is an essential component of good health care. It can provide timely, reliable and useful information to enable people to become an expert in managing their own health, in partnership with their healthcare providers.
11. **Criteria for antenatal and newborn screening and ethical controls**. Criteria for antenatal and newborn screening and ethical controls for other predictive testing need regular reconsideration in the light of changes in knowledge of disease causes, patient and support group awareness, and prevention possibilities.
12. **Recognition of specific problems of rare diseases in developing nations**. Governments should recognize the specific problems of rare diseases in developing nations and investigate ways in which screening, diagnosis, treatment and clinical training can be provided in aid programs or other arrangements.

## Conclusions

**Figure 1:**
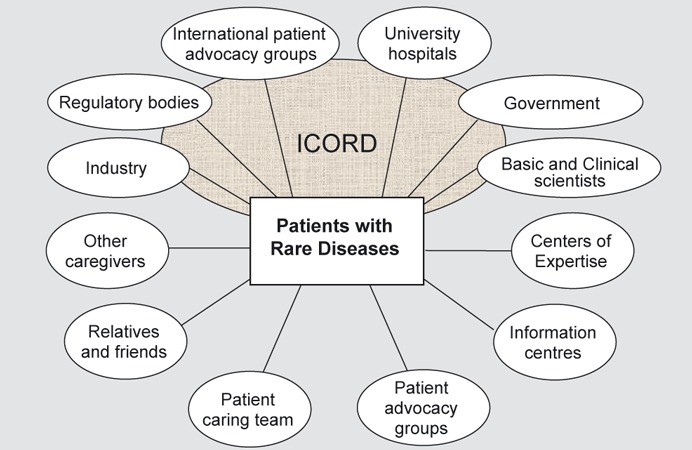
International Conference for Rare Disease and Orphan Drugs (ICORD) aims to facilitate contacts and networking among the many stakeholders involved in the health and welfare for patients with rare diseases. ICORD is a multidisciplinary non-profit society, drawing together members from healthcare services, patient advocacy groups, academia, medicine regulatory bodies, healthcare industry, and public policy agencies and organizations around the globe. In addition to the regular caring team, there are often other caregivers, supporting relatives and friends, and ideally a centre of expertise that can advice. To improve diagnostics, disease monitoring and treatment, both basic and clinical expertise is necessary, typically affiliated with university hospitals. Further information on diseases and treatments can be provided by information centres such as Orphanet (http://www.orpha.net). National governments and international health bodies have a central role on rare disease policies, and regulatory bodies review and support new drugs and medical devices developed by industry or academia. Patient advocacy groups can play a central role in many of the activities above.

The diagnosis, prevention and treatment needs of patients with most rare diseases and conditions remain largely unmet despite the significant efforts of many stakeholders (Fig. 1). For several selected rare diseases, remarkable basic research, clinical research and orphan products development activities have occurred leading to suitable treatments (1–6). However, more emphasis is required to support appropriate research and development activities leading to the development of prevention, diagnosis and treatments of rare diseases. Notably, clinical trials using already existing drugs and other treatments may be successful in finding new, affordable treatment strategies.

All countries are encouraged to implement specific research and development activities within their individual capabilities. Only when this has occurred will all patients around the world have equal access to necessary interventions to maximize the potential of every individual.
